# Prey‐soaking behavior in Iberian lynx

**DOI:** 10.1002/ecy.70364

**Published:** 2026-03-23

**Authors:** José Jiménez, Rafael Finat, Mario Fernández‐Tizón, Pedro Peiró, Javier Hernández‐Hernández, Antoni Margalida, Emilio Virgós

**Affiliations:** ^1^ Instituto de Investigación en Recursos Cinegéticos (IREC, CSIC‐UCLM‐JCCM) Ciudad Real Spain; ^2^ Finca el Castañar Toledo Spain; ^3^ Área de Biodiversidad y Conservación, Departamento de Biología, Geología, Física y Química Inorgánica Rey Juan Carlos University Madrid Spain; ^4^ Road Ecology Lab, Department of Biodiversity, Ecology and Evolution, Faculty of Biology Complutense University of Madrid Madrid Spain; ^5^ Pyrenean Institute of Ecology (CSIC) Jaca Spain

**Keywords:** animal culture, behavioral innovation, carnivore ecology, food handling, *Lynx pardinus*, social learning

In the dry heart of central Spain, a female Iberian lynx (*Lynx pardinus*) approaches a water trough—not to drink, but to immerse her freshly caught rabbit (*Oryctolagus cuniculus*) in water. To our knowledge, this unexpected prey‐soaking behavior has not been previously documented in wild carnivores. As we observed this behavior for the first time, we were prompted to investigate whether it may reflect a broader ecological or cognitive pattern among carnivores. Food‐washing or soaking behaviors are known in some primates, birds, and raccoons, but mostly in captive settings and among frugivorous or omnivorous species (Fiore et al., 2020; Lyall‐Watson, 1963; Zewald & Auersperg, 2023). In captivity, such behaviors have been documented in chimpanzees (*Pan troglodytes*), bonobos (*Pan paniscus*), gorillas (*Gorilla gorilla*), orangutans (*Pongo abelii*) (Allritz et al., 2013), wild boars (*Sus scrofa*) (Sommer et al., 2016), and Goffin's cockatoos (*Cacatua goffiniana*) (Zewald & Auersperg, 2023). Raccoons (*Procyon lotor*) also show similar behaviors in captivity (Goldman, 1950), possibly to enhance tactile sensitivity (Lyall‐Watson, 1963). In the wild, food‐washing behavior has only been reported in long‐tailed macaques (*Macaca fascicularis*) (Rosien et al., 2025).

Formally recorded through systematic camera trap monitoring, this novel behavior in Iberian lynx challenges conventional assumptions about prey‐handling in terrestrial carnivores. Carnivores are assumed to eat prey soon after killing and manipulate it mainly for tearing or caching (Allen et al., 2023), but not to alter its texture or use water. Purposeful soaking has not been reported. Our observation highlights the potential for overlooked behavioral diversity in wild felids and raises new questions about the ecological and evolutionary significance of such behaviors.

The Iberian lynx, hereafter referred to as “lynx,” is an endangered species that was on the brink of extinction in the 1980s. Thanks to an intensive conservation program primarily based on captive breeding and reintroduction efforts, its population has rebounded to an observed pre‐breeding population size of 1299 individuals in 2024 (Jiménez et al., 2025). Among the newly established populations, one of the largest is located in the Montes de Toledo region, in central Spain (Life Lynxconnect Team, 2024). During continuous monitoring with camera traps between 2014 and 2025 at the “El Castañar” estate, where five to six breeding females were established across 4000 ha over the study period, reaching nine adult females and eight adult males in 2025 (Figure 1; Appendix S1: Table S1), a previously undocumented behavior was observed at water troughs during June–August from 2020 onwards, involving the population's primary prey, the rabbit (*O. cuniculus*) (Delibes‐Mateos et al., 2007). Specifically, on 9 August 2020, a female lynx (*Naia*) was observed transporting a rabbit to a water trough. A similar event was recorded on 20 July 2023, involving another female (*Luna*) from an adjacent territory. On 20 August 2023, *Naia* was recorded for the first time actively immersing a rabbit in water for at least 60 s without releasing it, then retreating with the rabbit visibly soaked (see Figure 2 for a similar event). No feeding behavior was documented. Since 2020, when this behavior was first observed, until the present, eight prey‐soaking events were recorded involving five different females (four reproductive and one non‐breeder yearling) and five distinct water troughs (Figure 1). In four cases, prey immersion was directly observed. In the remaining four, the behavior was inferred from image sequences showing the lynx approaching the trough with prey and displaying soaking‐related postures, although the actual immersion was not captured (Appendix S1: Figure S1, Table S2).

**FIGURE 1:**
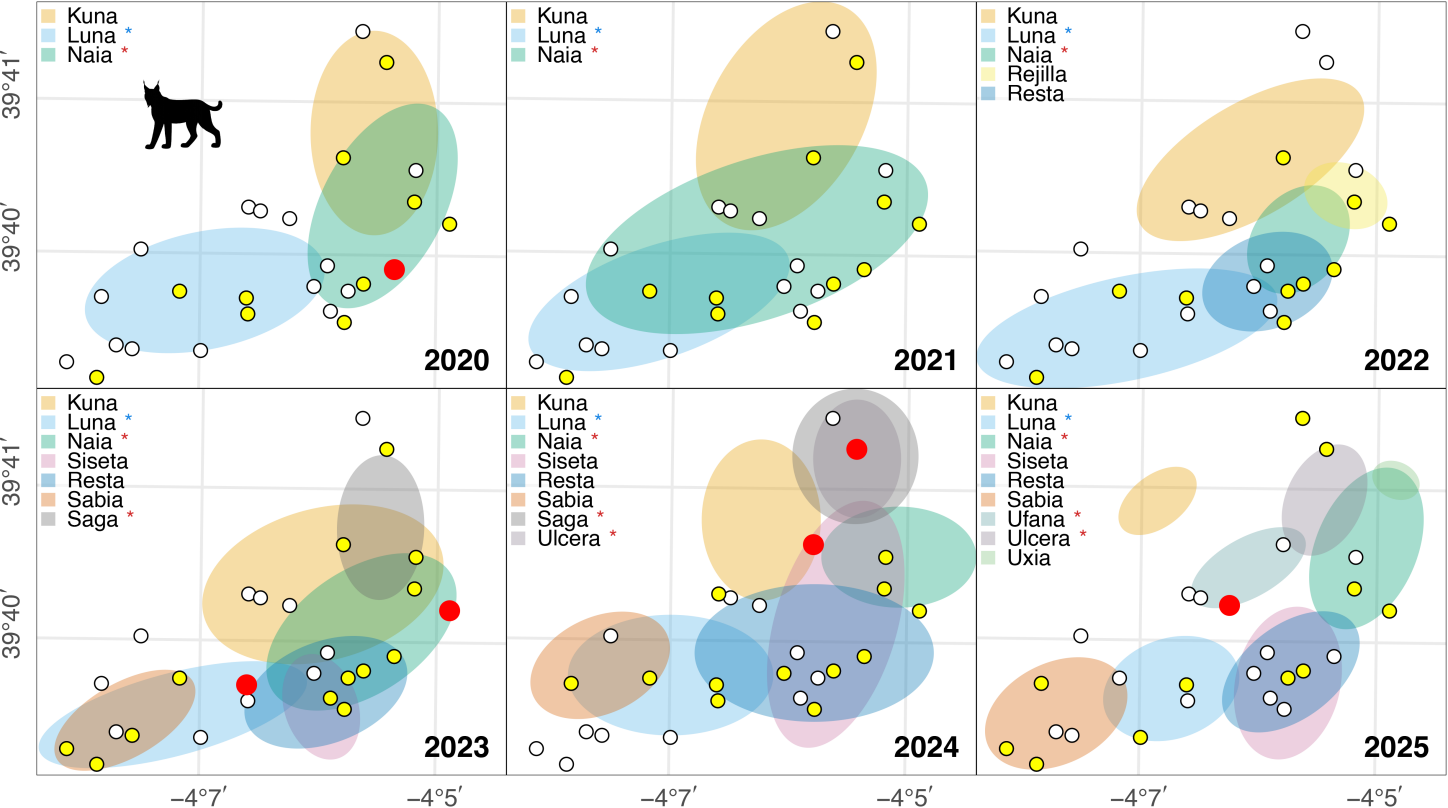
Observation areas of adult females in the study area from the first to the last recorded year of prey‐soaking behavior (2020–2025). The yearling female *Ulcera* was included in 2024 (see Appendix S1: Table S2). White dots indicate water troughs; yellow dots, troughs equipped with cameras; and red dots, troughs where prey‐soaking behavior was observed. To explore potential pathways for the spread of this behavior, particularly social transmission through kinship or spatial proximity, colored asterisks denote individuals with recorded prey‐soaking behavior: red for kinship relationships (see Appendix S1: Figure S2) and blue for spatial adjacency. Silhouette credit: José Jiménez.

**FIGURE 2:**
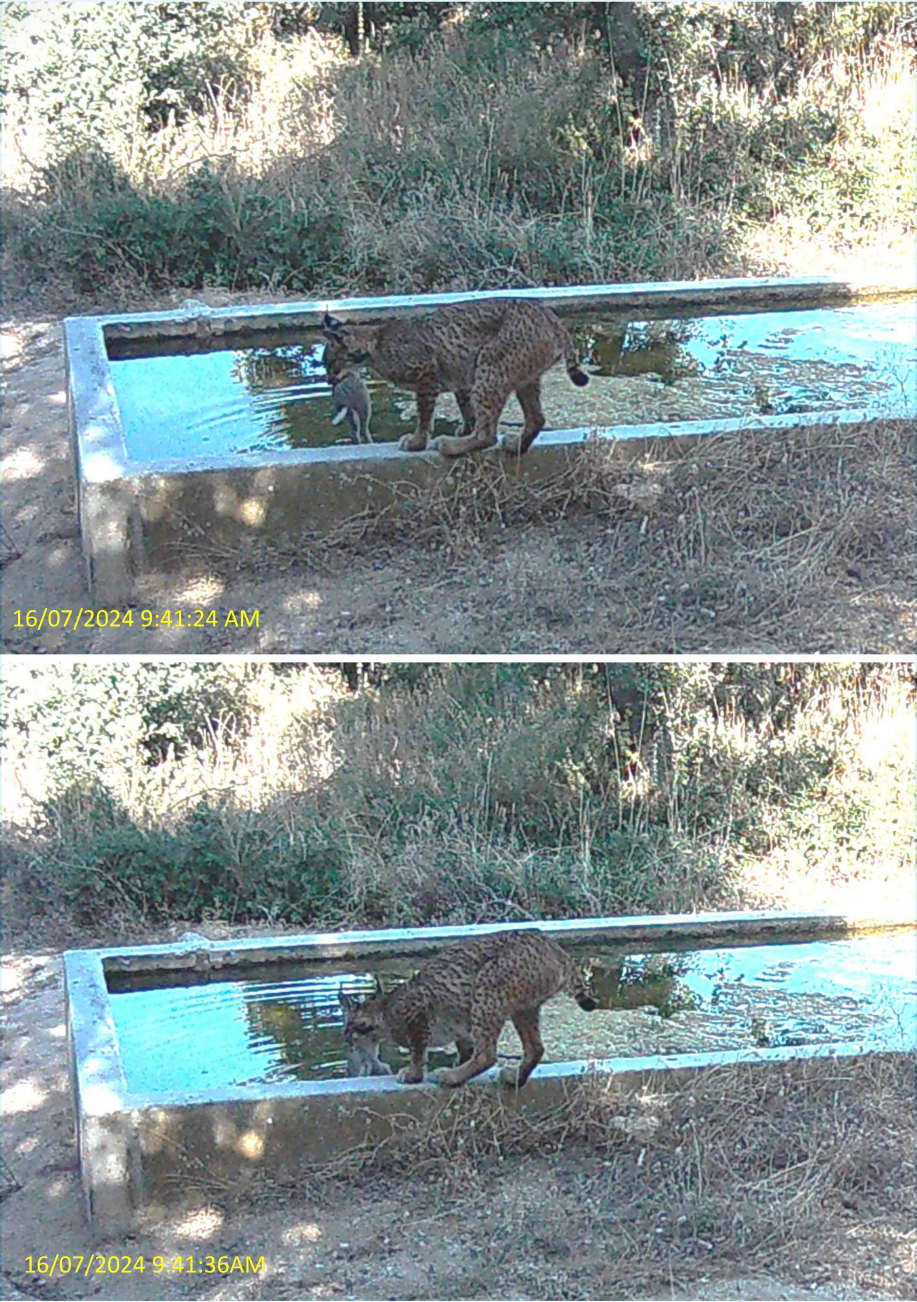
A female Iberian lynx soaking its prey—a rabbit—in water in Los Montes de Toledo (Spain) on 16 July 2024. We observed eight soaking events between 2020 and 2025. In the figure, images captured by camera traps have been cropped and brightness enhanced for clarity, without altering content. Original sequences are available in Appendix S1: Figure S1. Time annotations indicate seconds elapsed since the trigger event, as this information is not embedded in the photographs themselves. Images captured using motion‐triggered infrared camera traps deployed seasonally at water troughs. Image credit: Rafael Finat.

Notably, based on the camera trap records, ambient temperature during the observed events did not show extreme values or consistent patterns (see temperatures in Appendix S1: Figure S1) indicating that temperature is unlikely to act as an immediate trigger for this behavior, even though the seasonal context of late‐summer litters typically involves warmer and drier conditions. The timing of the observed behavior aligns with the lynx's peak activity periods, specifically between 08:00 and 10:00 in the morning, and from 23:00 to 01:15 at night (Appendix S1: Figure S1). This soaking behavior appears to be strictly localized to the Montes de Toledo region and has not been documented in other wild lynx populations or in captivity at breeding centers, where behavior is closely monitored through continuous video recordings (F. Villaespesa and A. Rivas, personal communication). All recorded events involved females, either in territories bordering those of other females previously recorded with this behavior, or in their descendants, where it was independently documented (Figure 1; Appendix S1: Figure S2). These events occurred between June and August, coinciding with the seasonal deployment of monitoring cameras at water troughs. Although the lynx is traditionally classified as a solitary carnivore, it exhibits a certain degree of sociability, particularly among related individuals (Sarmento et al., 2019). Observational and telemetry data from reintroduced populations have shown the formation of stable social cores and overlapping home ranges among females (Figure 1), suggesting that social interactions may play a role in territory establishment and cohesion (Rueda et al., 2021; Sarmento et al., 2019). The apparent transmission of prey‐soaking behavior within kin groups (Appendix S1: Figure S2) and spatially proximate individuals (Figure 1) may represent a rare case of socially mediated behavioral innovation in lynx, although direct evidence of learning remains unavailable. Its occurrence in a species traditionally considered solitary suggests a level of cultural potential rarely documented in wild carnivores (e.g., temporal niche switching in island foxes [*Urocyon littoralis*], Zhang et al., 2022; possible social learning among jaguars [*Panthera onca*], Raad et al., 2025; and resource‐induced behavioral shifts in pumas [*Puma concolor*], Serota et al., 2025). Recent studies on carnivore cognition further support the idea that even solitary species can exhibit socially mediated learning and behavioral innovation, especially in ecologically or socially relevant contexts (Benson‐Amram et al., 2023; Mazur & Seher, 2008). In felids, prey‐handling behavior has been shown to vary depending on prey type and environmental conditions, suggesting a degree of behavioral plasticity (Tallian et al., 2023).

We hypothesized that prey‐soaking behavior could serve a functional role, potentially facilitating hydration or easing the transition from milk to solid food during weaning, particularly in late‐season litters (Appendix S1: Table S2), which are typically born under warmer and drier conditions. As a complementary hypothesis, we considered that the behavior may represent a structured action shaped by maternal care and possibly influenced by maternal behavior (Klump, 2019; Tello‐Ramos et al., 2024; Thornton et al., 2010). To preliminarily assess functional mechanisms underlying prey‐soaking behavior, we conducted exploratory trials to assess how briefly immersing freshly killed rabbit carcasses affects internal temperature and water retention (see Figure 3). The experiment involved four rabbits: two dry (one in sun, one in shade) and two soaked (15 s in sun; 30 s in shade). Soaking times varied between sun and shade treatments to reflect natural field variability, rather than imposing standardized exposure. Our aim was to mimic realistic scenarios observed in the wild rather than test fixed immersion times. Our results showed that immersion slightly accelerated post‐mortem cooling, with a more pronounced effect under shaded conditions (Figure 3). Immersion also led to measurable water retention: after a 15‐s soak followed by exposure to direct sunlight, retained water dropped from 1.91% to just 0.3% of the body weight within 20 min. Under shaded conditions and a longer 30‐s soak (similar to those observed in the wild, see Appendix S1: Table S2), retained water decreased more slowly, from 5.14% to 3.7% after 40 min (Figure 3). Note that these conditions are not directly comparable and should be considered exploratory, non‐replicated, and illustrative only of potential functional effects of prey soaking. While cultural transmission remains a plausible alternative explanation, our trials suggest that prey soaking could serve a functional role beyond prey handling. Water retention in soaked carcasses suggests that female lynx might use this behavior to transport water, which could be beneficial during dry summer months; however, this remains hypothetical and warrants further study.

**FIGURE 3:**
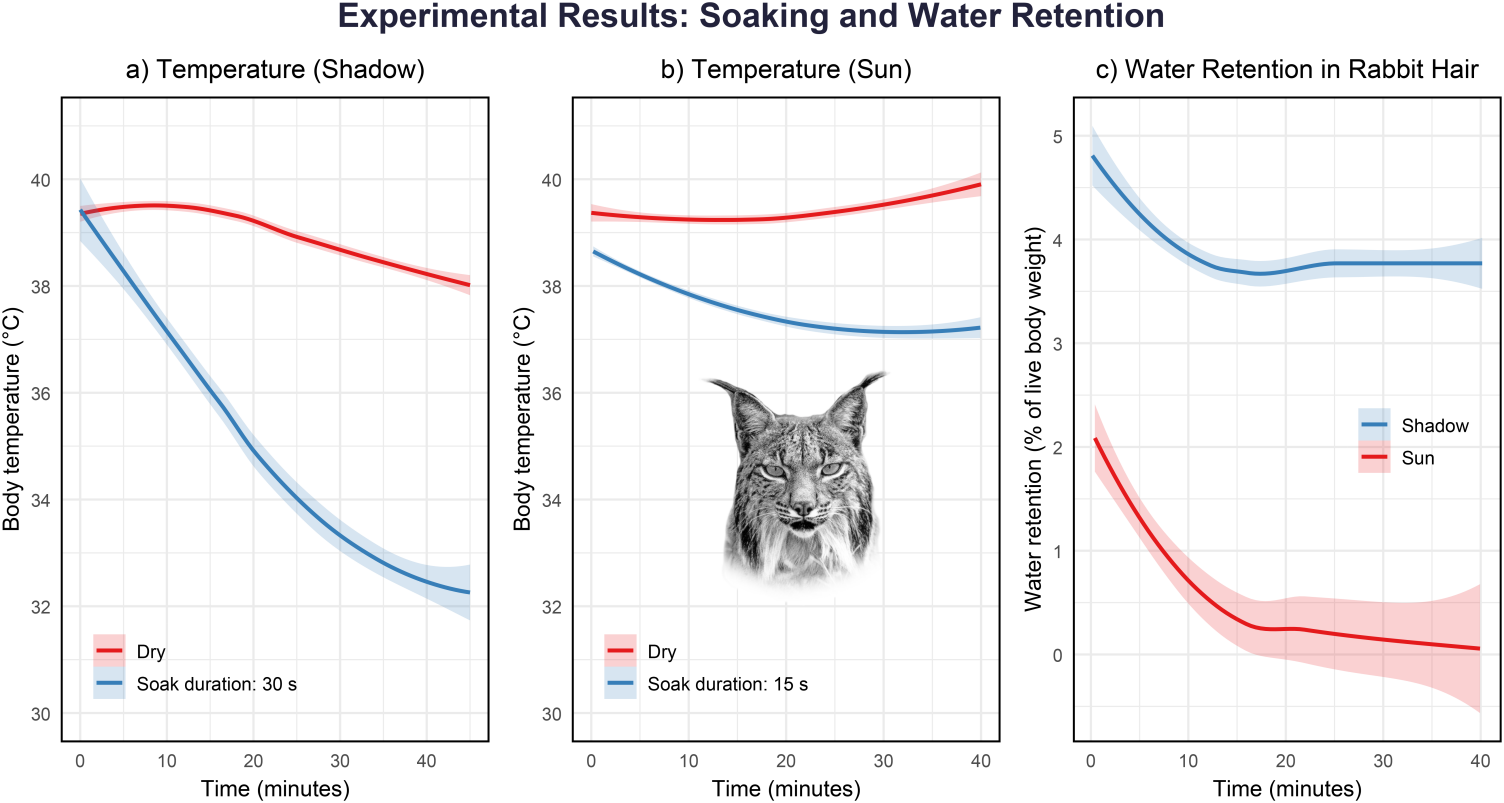
Body temperature change during 40 min for (a) one rabbit soaked for 15 s compared to one dry rabbit when kept in the shade, (b) one rabbit soaked for 30 s compared to one dry rabbit when kept in the sun, and (c) water retention change for one rabbit soaked for 30 s and kept in the shade compared to one rabbit soaked for 15 s and kept in the sun. Water retention was measured as percentage of live body weight. Air temperature was 21.0°C and water temperature was 21.2°C. Each line corresponds to a single rabbit, and the shaded area illustrates the variation observed during these measurements. Note that these conditions are not directly comparable and should be considered exploratory, non‐replicated, and illustrative only of potential functional effects of prey soaking. Illustration credit: José Jiménez.

Even in well‐monitored species like the Iberian lynx, unexpected behaviors continue to emerge (Tobajas et al., 2023) underscoring that natural history remains a vital tool for uncovering the nuances of animal life. Observations such as prey‐soaking behavior highlight the importance of maintaining open‐ended, long‐term behavioral monitoring, capable of revealing rare or novel traits that may otherwise go unnoticed. These findings highlight the value of integrating behavioral ecology into conservation programs (Van Overveld et al., 2020), whose monitoring should include not only demographic and population viability assessments, but also the identification of behavioral adaptations that may hold ecological or evolutionary significance. Understanding these behaviors can inform management strategies, especially in reintroduced or recovering populations where behavioral plasticity may influence both adaptation and long‐term persistence, especially in the context of climate change. Rapid adaptation through innovative behaviors is a key element of future adaptation to the fast change of environments under global warming (Shimada et al., 2010).

## AUTHOR CONTRIBUTIONS

José Jiménez, Rafael Finat, Emilio Virgós, Mario Fernández‐Tizón, and Antoni Margalida conceptualized the project and established the methodology. Rafael Finat carried out the fieldwork. Mario Fernández‐Tizón and Pedro Peiró carried out the experiments. José Jiménez led the writing of the manuscript, and all coauthors contributed to the final version of the manuscript.

## CONFLICT OF INTEREST STATEMENT

The authors declare no conflicts of interest.

## Supporting information


Appendix S1.

## Data Availability

Data (Jiménez, 2025) are available in Zenodo at: https://doi.org/10.5281/zenodo.17215002.
