# Supplementary material for: Prey‐soaking behavior in Iberian lynx
Source: Ecology. 2026 Mar 23;107(3):e70364. doi: 10.1002/ecy.70364 (PMC13008499; doi:10.1002/ecy.70364)
Supplement: Supplementary file 1 — Appendix S1. [file ECY-107-e70364-s001.pdf]

Appendix S1: Supplementary material

Prey-soaking behavior in Iberian lynx

José Jiménez, Rafael Finat, Mario Fernández-Tizón, Pedro Peiró, Javier Hernández-Hernández, Antoni Margalida, Emilio Virgós

Ecology

Table S1. Number of adult males (>2 years) in the study area (2020–2025).

Year	No. of males
2020	3
2021	2
2022	2
2023	4
2024	7
2025	8

Table S2. Summary of prey-soaking events in Iberian lynx recorded by camera traps between 2020 and 2025. Each row corresponds to a distinct soaking event (comprising several images; see Figure S1) and includes: event ID (ID), individual ID (Individual), observation date (Obs. Date), observation time (Obs. Time), ambient temperature (T, in °C), observed activity (Activity), either directly observed ('Soaking') or inferred from image context ('Apparently soaking'). For the duration of the soaking sequence ('Soak', in seconds), a minimum value was used when the final part of the sequence was missing, a maximum value when the entire sequence was available but exact timing could not be inferred, and NA when only a single image was available); age of the individual in years (Age), reproductive status (Repr.; Y = reproductive female, N = non-reproductive), and cub age in days (Cub age; NA if unknown or not applicable).

ID	Individual	Obs. Date	Obs. Time	T (ºC)	Activity	Age (years)	Soaking (seg)	Repr. (Y/N)	Cub age (days)
1	Naia	09/08/2020	20:49	39.0	Apparently soaking	3	NA	Y	NA
2	Luna	20/07/2023	8:05	25.0	Apparently soaking	8	> 240	Y	NA
3	Naia	20/08/2023	21:23	30.6	Soaking	6	> 60	Y	NA
4	Saga	02/06/2024	8:54	17.0	Apparently soaking	3	NA	Y	63
5	Naia	26/06/2024	22:20	24.0	Soaking	7	> 240	Y	60
6	Naia	09/07/2024	1:10	22.0	Soaking	7	> 60	Y	73
7	Ulcera	16/07/2024	9:41	20.0	Soaking	1	< 60	N	NA
8	Ufana	25/06/2025	9:25	25.0	Apparently soaking	2	< 60	Y	NA

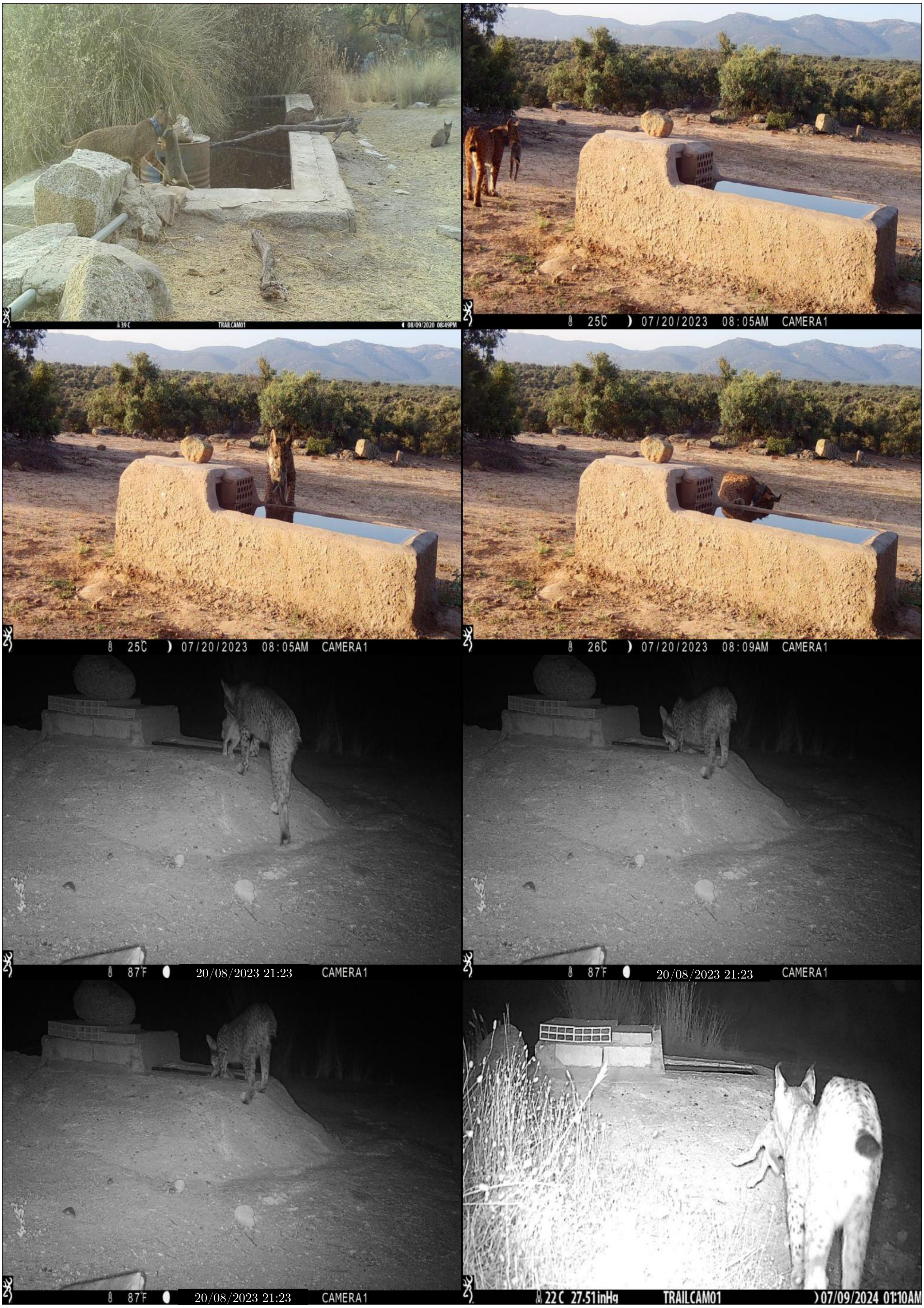
39C
TRAILCAM01
08/09/2020 08:49PM
25C 07/20/2023 08:05AM CAMERA1
25C 07/20/2023 08:05AM CAMERA1
26C 07/20/2023 08:09AM CAMERA1
87°F 20/08/2023 21:23 CAMERA1
87°F 20/08/2023 21:23 CAMERA1
87°F 20/08/2023 21:23 CAMERA1
22C 27.51inHg TRAILCAM01 07/09/2024 01:10AM

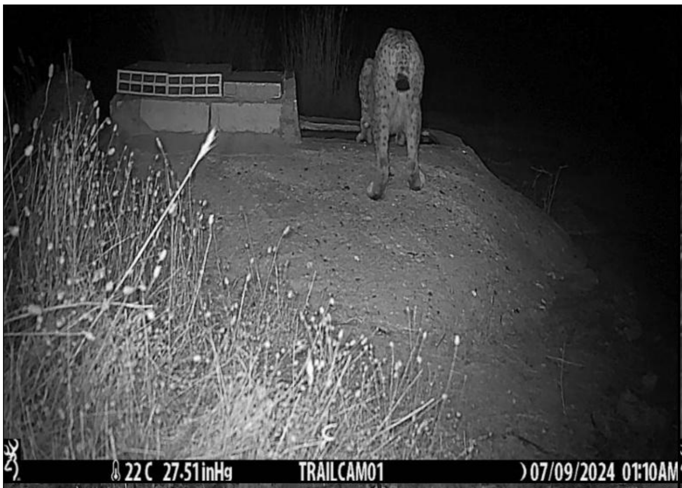
22 C 27.51 inHg
TRAILCAM01
07/09/2024 01:10AM

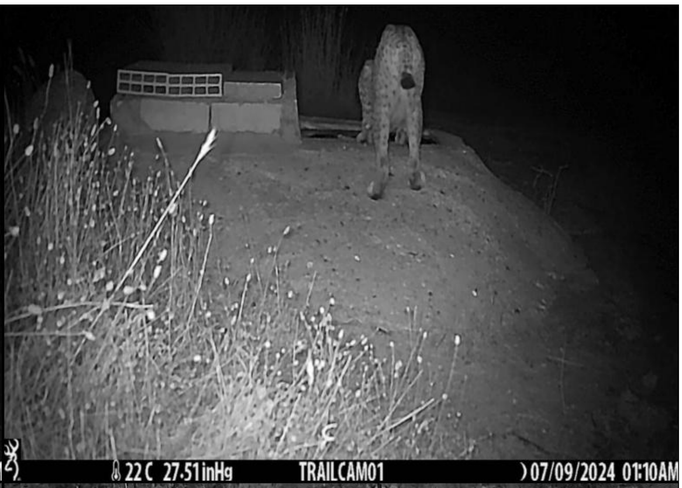
22 C 27.51 inHg
TRAILCAM01
07/09/2024 01:10AM

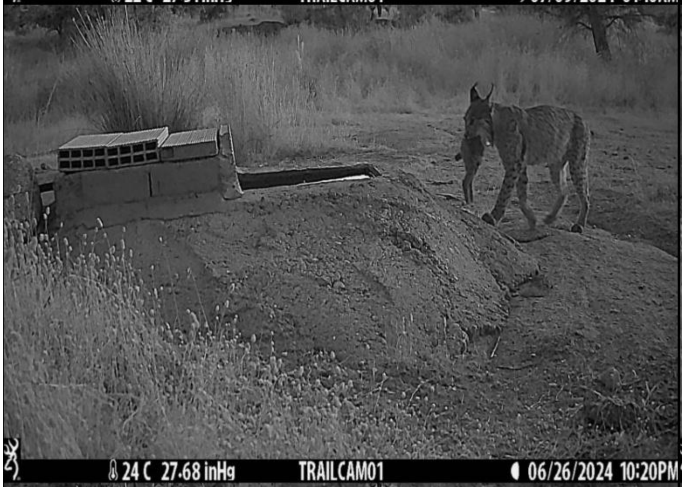
24 C 27.68 inHg
TRAILCAM01
06/26/2024 10:20PM

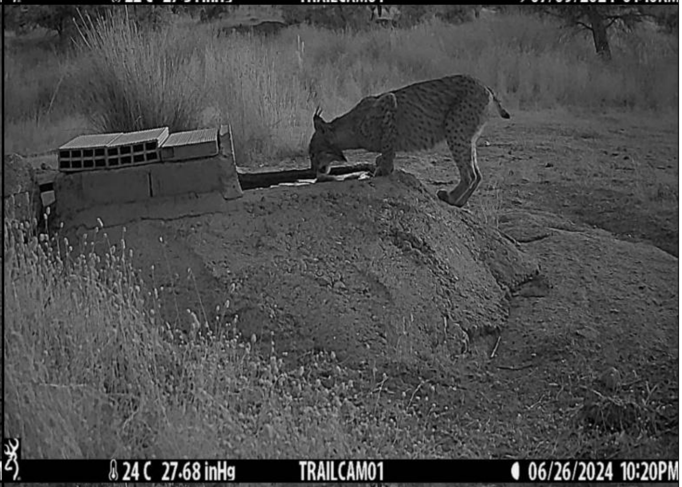
24 C 27.68 inHg
TRAILCAM01
06/26/2024 10:20PM

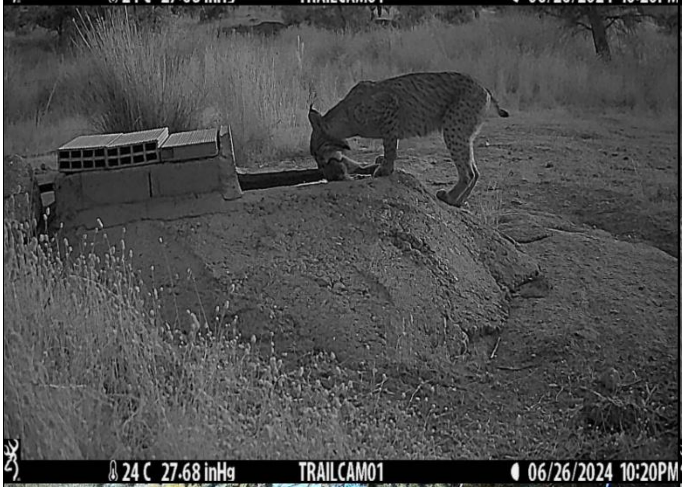
24 C 27.68 inHg
TRAILCAM01
06/26/2024 10:20PM

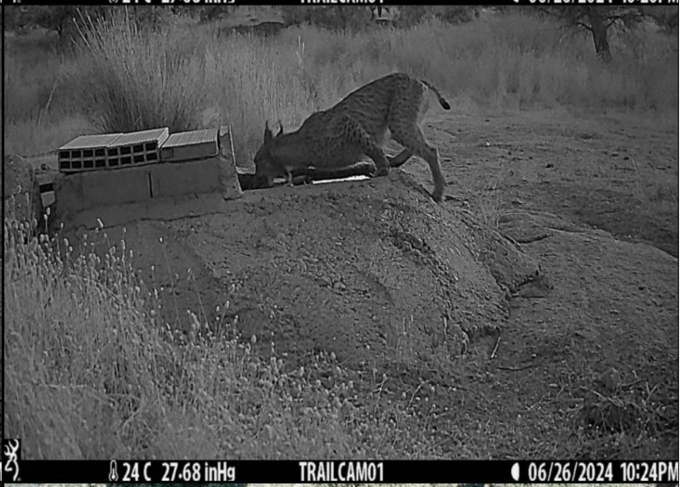
24 C 27.68 inHg
TRAILCAM01
06/26/2024 10:24PM

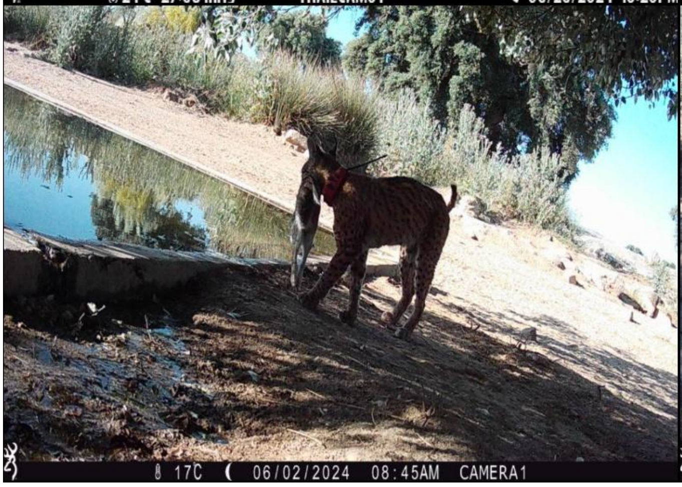
17C
06/02/2024 08:45AM CAMERA1

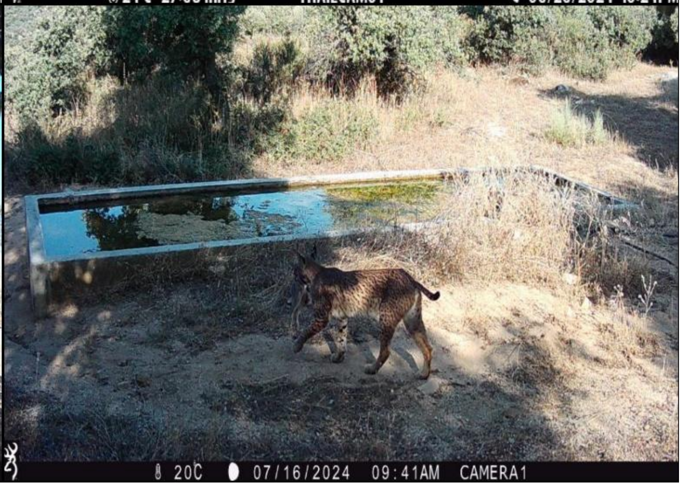
20C
07/16/2024 09:41AM CAMERA1

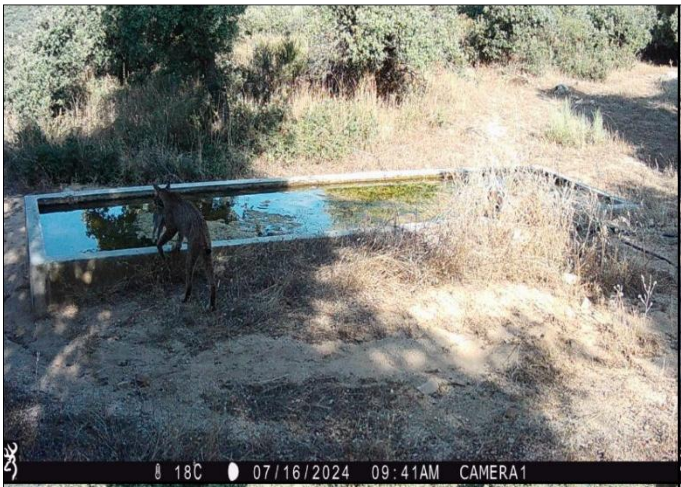
18°C 07/16/2024 09:41AM CAMERA1
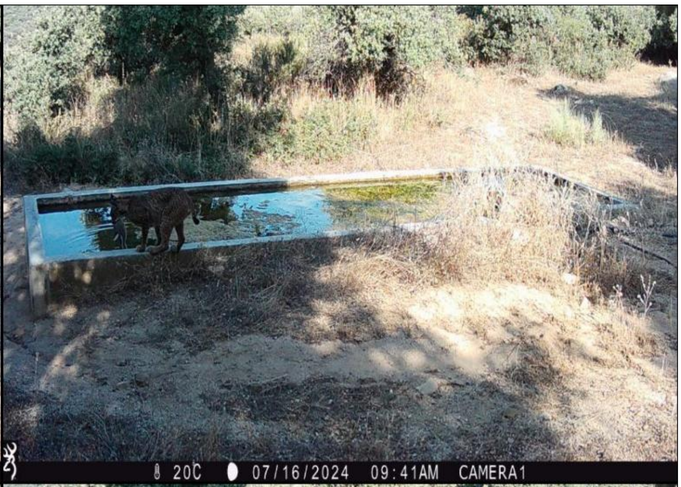
20°C 07/16/2024 09:41AM CAMERA1
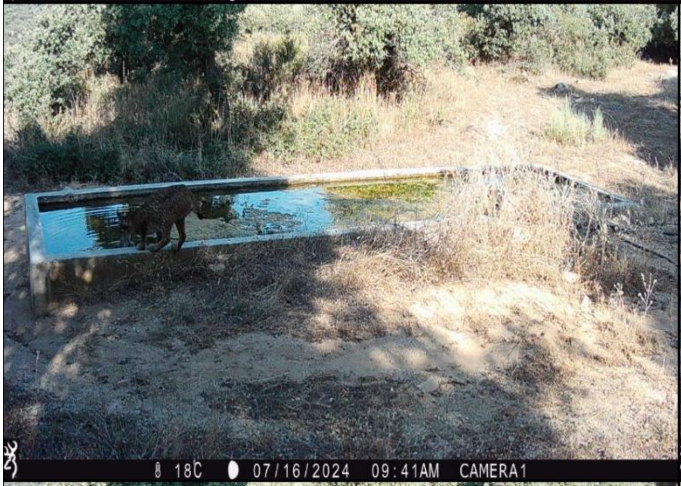
18°C 07/16/2024 09:41AM CAMERA1
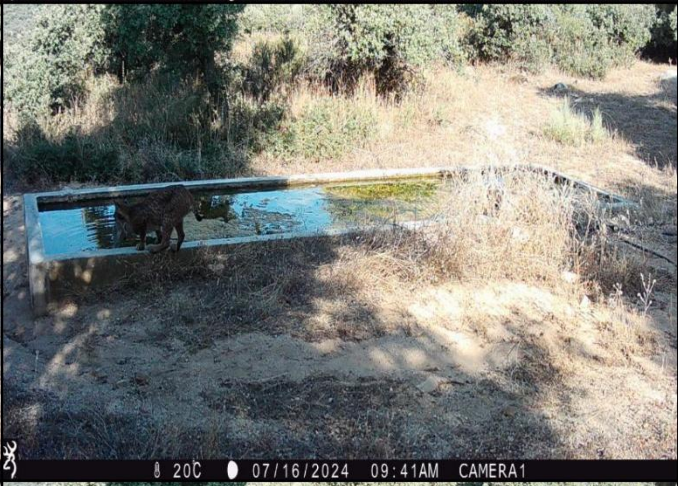
20°C 07/16/2024 09:41AM CAMERA1
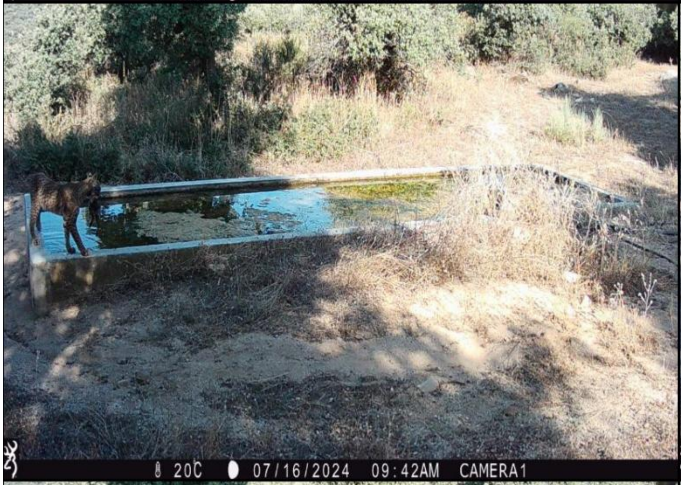
20°C 07/16/2024 09:42AM CAMERA1
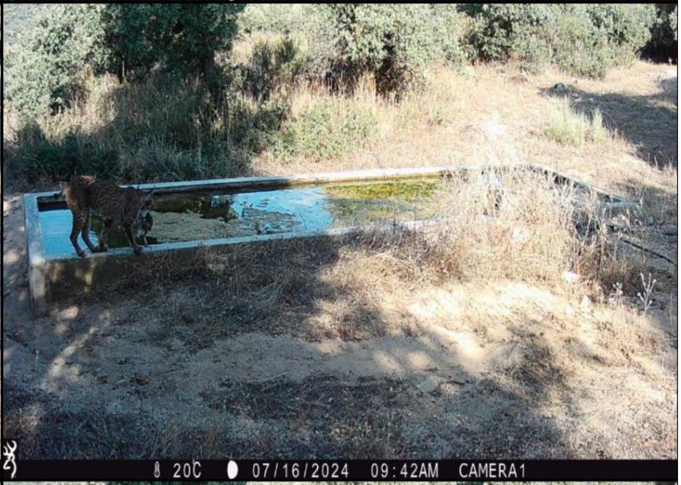
20°C 07/16/2024 09:42AM CAMERA1
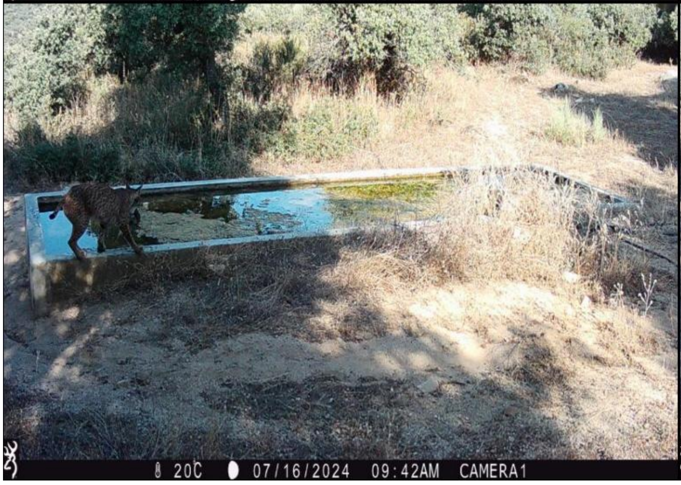
20°C 07/16/2024 09:42AM CAMERA1
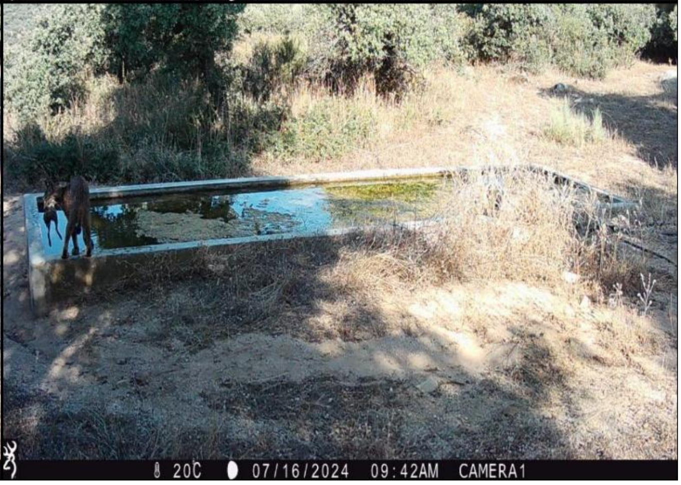
20°C 07/16/2024 09:42AM CAMERA1

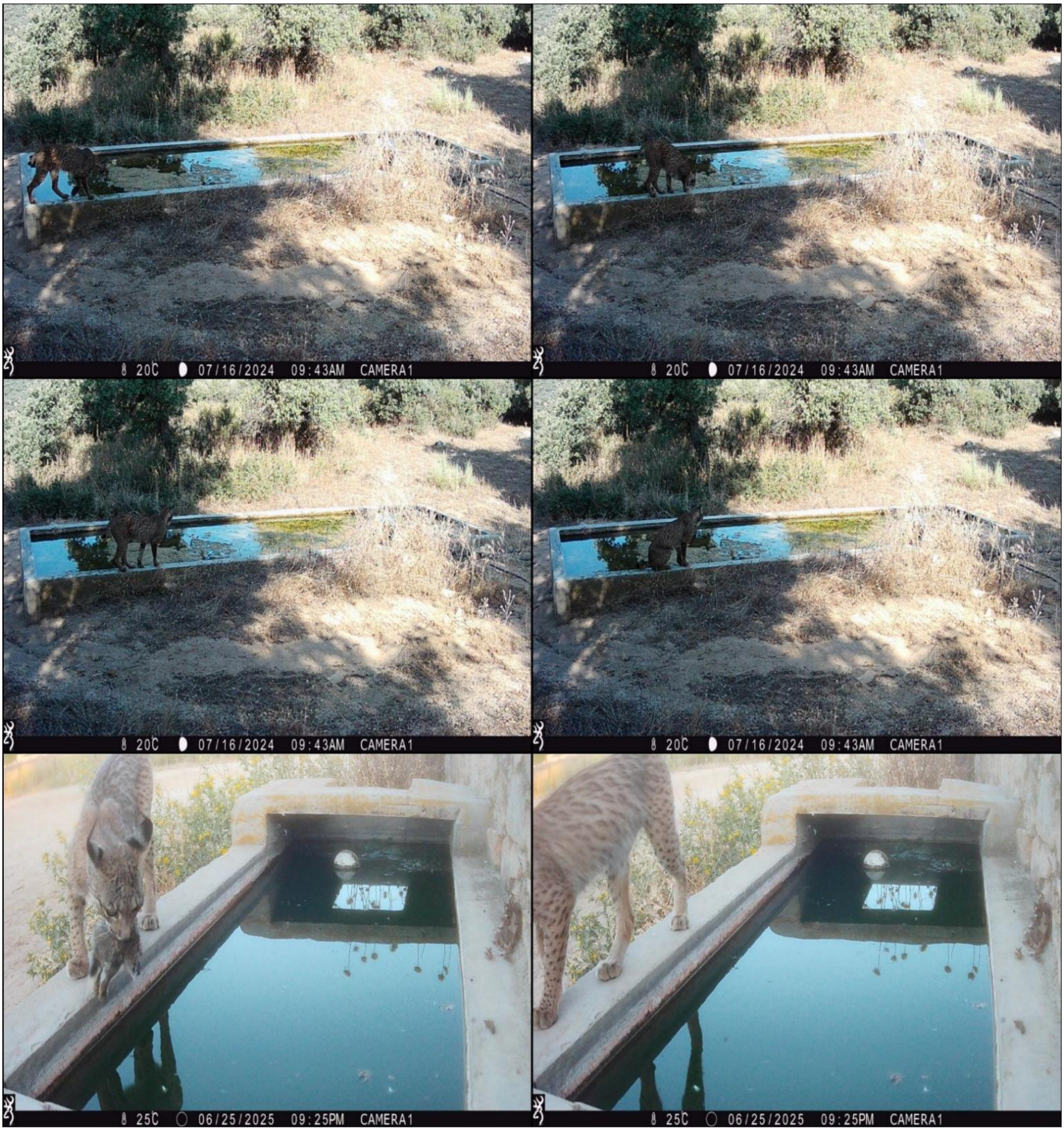

Figure S1. Original screenshots of soaking events recorded by camera traps (2020–2025) in Los Montes de Toledo (Spain). Note that the photographs dated 20/08/2023 have a date and time overprinted manually, as the original metadata was incorrect due to misconfigured camera settings. Image credit: Rafael Finat.

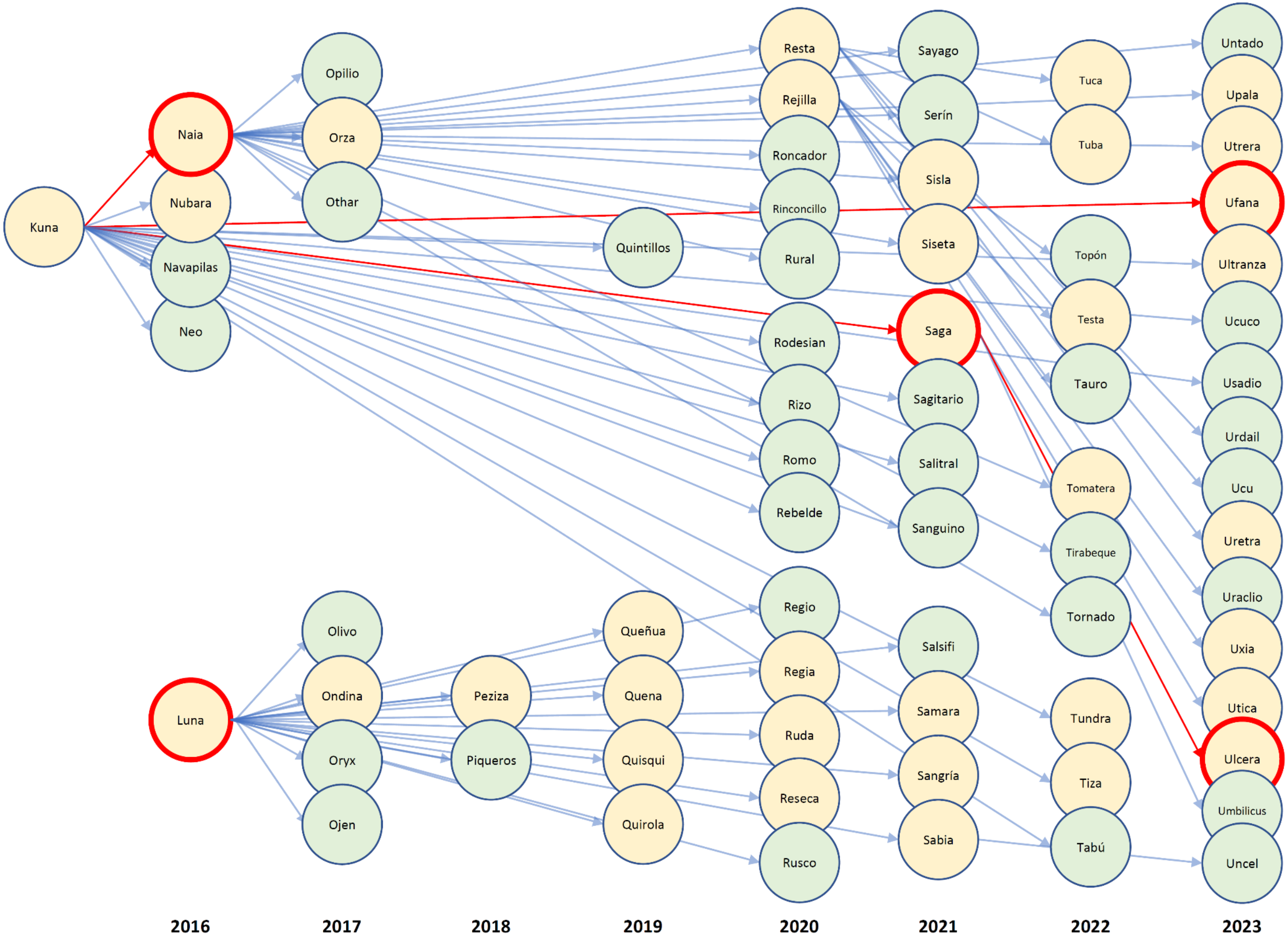

Figure S2. Genealogy diagram of lynx individuals observed displaying prey-soaking behavior (highlighted in red). Red arrows indicate maternal relationships among these individuals, connecting each female to her offspring. Kuna and Luna originated from captive breeding centers and were introduced at one years of age in 2014 and 2015, respectively.
